# Gene expression in nontumoral liver tissue and recurrence-free survival in hepatitis C virus-positive hepatocellular carcinoma

**DOI:** 10.1186/1476-4598-9-74

**Published:** 2010-04-09

**Authors:** Masato Tsuchiya, Joel S Parker, Hiroshi Kono, Masanori Matsuda, Hideki Fujii, Ivan Rusyn

**Affiliations:** 1 Department of Environmental Sciences and Engineering, Gillings School of Global Public Health, University of North Carolina, Chapel Hill, NC, USA; 2 Curriculum in Genetics, School of Medicine, University of North Carolina, Chapel Hill, NC, USA; 3 First Department of Surgery, University of Yamanashi, Chuo, Yamanashi Prefecture, Japan

## Abstract

**Background:**

The goal of this study was to understand gene expression signatures of hepatocellular carcinoma (HCC) recurrence in subjects with hepatitis C virus (HCV) infection. Recurrence-free survival (RFS) following curative resection of HCC in subjects with HCV is highly variable. Traditional clinico-pathological endpoints are recognized as weak predictors of RFS. It has been suggested that gene expression profiling of HCC and nontumoral liver tissue may improve prediction of RFS, aid in understanding of the underlying liver disease, and guide individualized patient management. Frozen samples of the tumors and nontumoral liver were obtained from 47 subjects with HCV-associated HCC. Additional nontumoral liver samples were obtained from HCV-free subjects with metastatic liver tumors. Gene expression profiling data was used to determine the molecular signature of HCV-associated HCC and to develop a predictor of RFS.

**Results:**

The molecular profile of the HCV-associated HCC confirmed central roles for MYC and TGFβ1 in liver tumor development. Gene expression in tumors was found to have poor predictive power with regards to RFS, but analysis of nontumoral tissues yielded a strong predictor for RFS in late-recurring (>1 year) subjects. Importantly, nontumoral tissue-derived gene expression predictor of RFS was highly significant in both univariable and multivariable Cox proportional hazard model analyses.

**Conclusions:**

Microarray analysis of the nontumoral tissues from subjects with HCV-associated HCC delivers novel molecular signatures of RFS, especially among the late-recurrence subjects. The gene expression predictor may hold important insights into the pathobiology of HCC recurrence and *de novo *tumor formation in cirrhotic patients.

## Background

Hepatocellular carcinoma (HCC) is the fifth most common cancer worldwide by annual incidence and the third leading cause of cancer death [1]. Wide geographic variation in age-adjusted incidence and death rates is well recognized [2]. Most alarming is the fact that age-adjusted incidence and death rates for cancer of the liver and intrahepatic bile duct show a statistically significant increasing trend in the past three decades in the USA and many other countries, even though other major cancers are on a decline [3]. This rise is being attributed, at least in part, to an increase in incidence of hepatitis C virus (HCV) infections and non-alcoholic steatohepatitis, pathological states that are also growing in the US population [4,5]. The resistance of HCC to existing treatments and the lack of biomarkers for early detection make it one of the deadliest cancers. Surgical resection, liver transplantation, and ablation by radiofrequency or ethanol injection are now conventional therapies at early disease stages. Even with these options, survival at 5 years is poor and ranges between 50% and 70% [6].

One of the key reasons for poor long-term survival in HCC is high incidence of recurrence, a complication that cannot be prevented effectively by new and existing therapies [7,8]. Many clinico-pathological features, such as tumor size, number of tumors in liver, capsule state, cell differentiation, venous invasion, and the extent of intrahepatic spreading are commonly used in clinical diagnosis as predictive risk factors for HCC recurrence and health prognosis in patients [9,10]. However, the prospective utility of these attributes for predicting recurrence-free survival (RFS) may be more limited as HCC is being diagnosed at earlier stages.

Several research groups have performed gene expression profiling of both tumor and nontumoral specimens and identified gene signatures of recurrence-free [11], or overall survival [12] in HCC patients who undergo tumor resection. These studies carry an exciting potential to open the field to more selective chemoprevention as a follow-up to surgical interventions since patients with greatest risk of death or recurrence can be potentially identified [12] and/or individualized therapies may be devised based on the molecular profiles of poor-prognosis markers of HCC [13,14]. At the same time, the issues of validation of the molecular signatures, the utility of target-gene list strategy for predicting early- versus late-recurrence, and the value of these studies for understanding pathobiology of the underlying liver disease in patients with HCC are still open for consideration [15].

Thus, our study focused on a well-defined cohort of HCC patients who were HCV positive (but free of hepatitis B virus) and underwent tumor resection to conduct a comparative microarray-based gene expression profiling of tumor and nontumoral specimens. We report the molecular network signatures of HCV-associated HCC, as well as the outcome of the analysis of the predictive value of gene expression in tumor- and nontumoral tissue-derived samples. This study was not only successful in developing a highly predictive RFS signature for late recurrence from nontumoral samples, but also shows that this signature can be used for biological interpretation of the liver disease leading to recurrence.

## Results and Discussion

### Clinico-pathological markers of RFS in HCV-associated HCC

This study evaluated specimens (frozen tumor and/or nontumoral tissue) collected during liver tumor resection in (i) subjects with solitary tumors in liver that were a result of metastasis from other organs, and (ii) subjects manifesting HCC in combination with chronic HCV infection and absence of known tumors at other sites. Platelet count, alanine aminotransferase and fibrosis score were significantly different between the groups (Table 1). The HCC cohort was subdivided into nontumoral and tumor groups for the purposes of molecular analysis (see below). No differences between these two groups, or between each group and the entire cohort, exist based on the available clinical data. The univariable analysis of the prognostic power for various clinico-pathological measurements available in this study showed that tumor stage, diameter and, to a lesser degree, multiplicity were statistically significant in predicting the length of RFS in subjects with HCV-associated HCC (Table 2). Other characteristics were not significant, including the type of the surgical procedure performed (e.g., lobular or segmental/partial hepatectomy). These results are consistent with the reports in the literature [16].

**Table 1:** Clinical characteristics of patients.

Variables	HCC Cohort (N = 47)	Nontumoral HCC cohort (N = 44)	Tumor HCC cohort (N = 43)	Control liver cohort (metastatic liver tumors, N = 8)
Age	66.5 ± 7.8	66.4 ± 7.9	66.5 ± 7.9	60.0 ± 13.5
Gender				
Male	35 (74.4%)	32 (72.7%)	32 (74.4%)	4 (50.0%)
Female	12 (25.5%)	12 (27.3%)	11 (25.6%)	4 (50.0%)
Viral infection status				
HBV infection	-	-	-	-
HCV infection	+	+	+	-
Liver resection procedure				
Lobular/extended lobular	5	5	5	N/A
Segmental/partial	42	39	38	
Platelet count (10^4^/μl)	12.9 ± 4.3	13.0 ± 4.2	12.8 ± 4.2	19.3+3.6*
Alanine aminotransferase (IU/l)	51.0 ± 31.7	51.0 ± 32.0	51.7 ± 33.1	19.7+7.7*
Total bilirubin (mg/dl)	0.80 ± 0.34	0.76 ± 0.30	0.80 ± 0.35	0.85+0.37
Prothrombin time (%)	78.3 ± 13.1	78.3 ± 12.9	78.1 ± 13.3	91.1+14.5
Indocyanine green (%)	18.4 ± 8.3	18.5 ± 8.3	18.3 ± 8.4	11.0+5.3
α-Fetoprotein (ng/ml)	319 ± 924	339 ± 952	345 ± 962	N/A
Tumor diameter (cm)	2.90 ± 1.30	2.97 ± 1.35	2.95 ± 1.37	N/A
Number of tumors	1.30 ± 0.50	1.32 ± 0.52	1.30 ± 0.51	N/A
Portal vein invasion				
Present	5 (10.6%)	5 (11.4%)	5 (11.6%)	N/A
Absent	42 (89.4%)	39 (88.6%)	38 (88.4%)	N/A
Tumor stage				
I	9 (19.1%)	9 (20.5%)	8 (18.6%)	N/A
II	25 (53.2%)	23 (52.3%)	24 (55.8%)	N/A
III	12 (25.5%)	11 (25.0%)	10 (23.3%)	N/A
IV	1 (2.1%)	1 (2.3%)	1 (2.3%)	N/A
Tumor differentiation				
Well differentiated	8 (17.0%)	8 (18.2%)	6 (14.0%)	N/A
Moderately differentiated	31 (66.0%)	28 (63.6%)	29 (67.4%)	N/A
Poorly differentiated	8 (17.0%)	8 (18.2%)	8 (18.6%)	N/A
Fibrosis score				
F0	0 (0%)	0 (0%)	0 (0%)	8 (100%)*
F1	3 (6.4%)	3 (6.8%)	3 (7.0%)	0 (0%)
F2	6 (12.8%)	5 (11.4%)	6 (14.0%)	0 (0%)
F3	14 (29.8%)	14 (31.8%)	12 (27.9%)	0 (0%)
F4	24 (51.1%)	22 (50.0%)	22 (51.2%)	0 (0%)

Data shown are mean ± standard deviation. N/A, data is not available. Asterisk(*) denotes statistical difference from other groups (ANOVA, p < 0.05).

**Table 2:** Univariate analysis of clinico-pathological prognostic markers associated with recurrence-free survival in patients with HCC.

	Nontumoral HCC Cohort (N = 44)			Tumor HCC Cohort (N = 43)		
	
	Risk ratio	95% CI	P-value	Risk ratio	95% CI	P-value
**Tumor stage**	**2.48**	**1.39-4.51**	**0.002**	**2.54**	**1.41-4.70**	**0.002**
**Tumor diameter**	**1.59**	**1.17-2.09**	**0.005**	**1.65**	**1.19-2.24**	**0.004**
**Number of tumors**	**2.41**	**1.17-4.68**	**0.02**	**2.10**	**0.99-4.19**	**0.05**
Platelet count	0.95	0.87-1.03	0.22	0.97	0.89-1.06	0.53
Sex [male]	1.44	0.67-3.43	0.36	1.62	0.74-4.08	0.24
Indocyanine green	1.02	0.97-1.06	0.40	1.01	0.97-1.05	0.56
Prothrombin time	0.99	0.97-1.02	0.45	0.99	0.97-1.02	0.40
Portal vein invasion [present]	1.41	0.33-4.06	0.59	1.42	0.34-4.08	0.59
Liver resection procedure [lobular]	1.14	0.62-1.82	0.65	1.13	0.61-1.81	0.66
Total Bilirubin	1.26	0.42-3.24	0.66	1.35	0.50-3.19	0.54
Fibrosis score	0.96	0.65-1.49	0.86	0.92	0.63-1.38	0.67
Tumor differentiation [moderate-poor]	1.08	0.47-2.93	0.86	0.84	0.35-2.49	0.73
Age	1.00	0.96-1.05	0.90	1.02	0.97-1.07	0.53
Alanine aminotransferase	1.00	0.99-1.01	0.96	1.00	0.99-1.01	0.94
α-Fetoprotein	1.00	1.00-1.00	0.99	1.00	1.00-1.00	0.90

Patients with HCV-associated HCC were divided into two cohorts based on the availability of mRNA samples. Univariate Cox proportional hazard model analysis was used to assess relative risk for each clinical variable. Relative risk >1 identifies shortened recurrence-free survival. Significant variables are highlighted in bold.

### Molecular profiling of HCV-associated HCC

**Figure 1:**
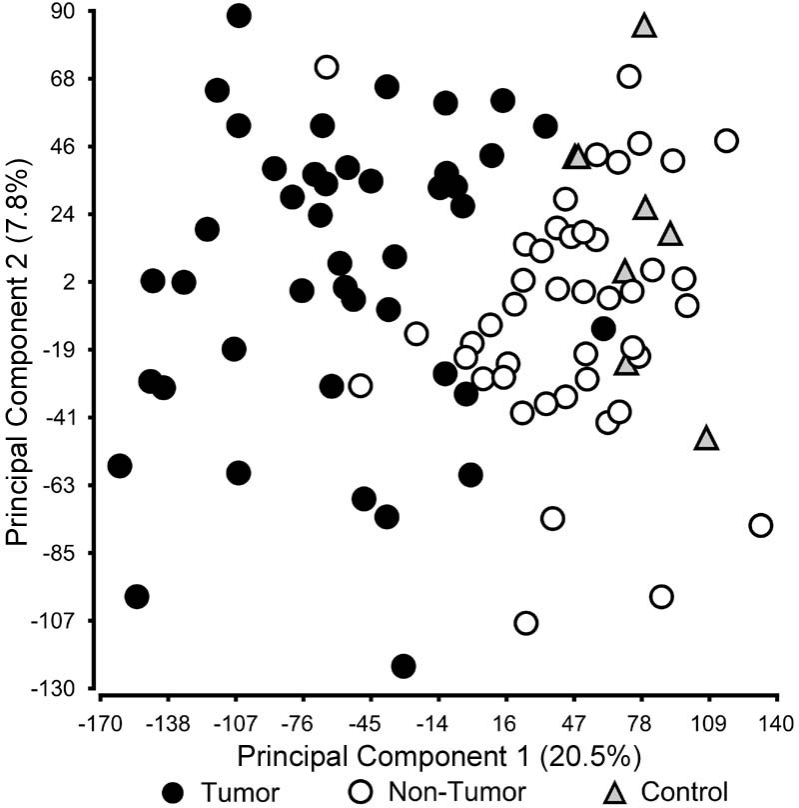
**Principal components analysis of the liver tumoral and non-tumoral transcriptome**. Global gene expression (25,073 genes) data of the samples from HCC (black circle), surgical margin-derived nontumoral samples (white circle), and control samples (nontumoral samples from patients with metastatic liver tumors; black triangle) was visualized using principal components analysis.

The importance of defining liver tumor biology that may aid in the development of new screening and treatment stratification programs to refine diagnosis and improve patient outcome is well recognized [15]. Thus, our first step was to evaluate gene expression data (25,073 transcripts) collected on individual tumor and nontumoral samples. Principal components analysis (Genomics Suite; Partek Inc., St. Louis, MO) was used to examine global variation in transcript abundance (Figure 1). The unsupervised analysis displayed separation of the samples between HCC tumor samples and nontumoral samples from subjects with HCC and metastatic liver tumors indicating that gene signatures indicative of HCC tumor biology may be explored.

To evaluate the biological features of HCV-associated HCC we compared gene expression profiles of tumors and control (nontumoral tissue from metastatic liver tumor) samples. The analysis revealed 155 up- and 1,248 down-regulated genes whose expression levels were significantly different (FDR = 0). Next, functional pathway analysis was performed using significant genes (Additional file 1, Tables S2-3). Several pathways were identified as significantly up- or down-regulated in HCV-associated HCC. Interestingly, the top-ranked network composed of up-regulated genes includes TGFβ1 and MYC as hubs (Additional file 2, Figure S1). Indeed, it has been suggested that chronic inflammation associated with HCV infection in liver facilitates TGFβ signaling cascade involving c-Jun N-terminal kinase and plasminogen activator inhibitor 1 which facilitates fibrogenesis, promotes liver cirrhosis and increases the risk of development of HCC [17]. In addition, gene expression-based comparison between dysplastic nodules and early hepatocellular carcinomas identified the MYC oncogene as a plausible driver gene for malignant transformation of dysplastic nodules [18]. Thus, our data provides independent confirmation of and strengthens the evidence [19] for the key roles of TGFβ1 and MYC in the progression of HCC in patients with HCV.

### Tumor-derived gene expression profiles and recurrence-free survival in HCV-associated HCC

Recurrence of HCC is thought to arise due to intra-hepatic metastasis, or the development of a second primary tumor in a liver that is cirrhotic due to concomitant disease [20]. The molecular signatures of the tumor and its surrounding tissue, obtained through gene expression, proteomics or other means of profiling, are expected to reflect the causes of recurrence. Thus, an important challenge is to develop better understanding of the biological characteristics of the tumor and the surrounding tissue and to use this information to predict the outcomes.

Indeed, gene expression profiling has been instrumental in tackling these challenges and it has been shown that microarray analysis of the tumor samples can be used for prediction of clinical outcomes in subjects with HCC [21-23]. At the same time, others have reported no success with tumor-based transcription profiling [12]. Despite the fact that many clinical variables from tumors such as number of tumors, tumor stage, and tumor size were significantly associated with recurrence (Table 2), univariable feature selection with Cox scores did not identify robust markers of tumor recurrence in the context of leave-one-out cross-validation either in the entire cohort or in the sub-cohort of the early (<1 year) recurring subjects (data not shown). Possible reasons for our inability to create a predictor with tumor samples alone may include, among other factors, small sample size compounded by the considerable heterogeneity of gene expression between tumor samples (Additional file 2, Figure S2). However, our result is similar to the observations of Hoshida *et al*. [12] who also were unable to establish a tumor-derived predictor even with a much larger (hundreds of subjects) cohort. Although these results do not negate the value of tumor-derived expression profiling in predicting the outcomes of HCC, the data suggest that, at least in the cohort of HCV-positive HCC, tumor profiling alone may have limited value with regards to RFS.

Nonetheless, to further characterize the pathobiology of HCC recurrence we applied the gene set analysis algorithm to tumor-derived gene expression data in order to determine whether biologically-relevant gene sets that correlate with RFS exist. There were 1,497 gene sets based on 25,073 transcripts and a Cox score was generated for each gene set. Based on the nearest template prediction and leave-one-out cross-validation (i.e., independent prediction of each sample), several top-ranked (based on Cox score) gene sets were found to be predictive of tumor recurrence (Additional file 2, Figure S3). The gene set analysis has great value for assessment of the molecular profiles of the aggressively recurring tumors and may improve our understanding of the underlying biology that may be associated with the poor outcomes. Indeed, the most predictive gene set-based survival signature from HCC samples (Additional file 1, Table S4) contains a number of known key cancer-promoting pathways, such as mTOR [24], TGFβ [17], DNA damage response [25], hypoxia [26], histone deacetylase [27], and MET [28]. This data suggests that key tumor-related genes may not only be involved in tumorigenesis, but may also be important for recurrence and thus can be used for selection of the subjects with poor prognosis.

### Nontumoral tissue-derived gene expression profiles and recurrence-free survival in HCV-associated HCC

**Figure 2:**
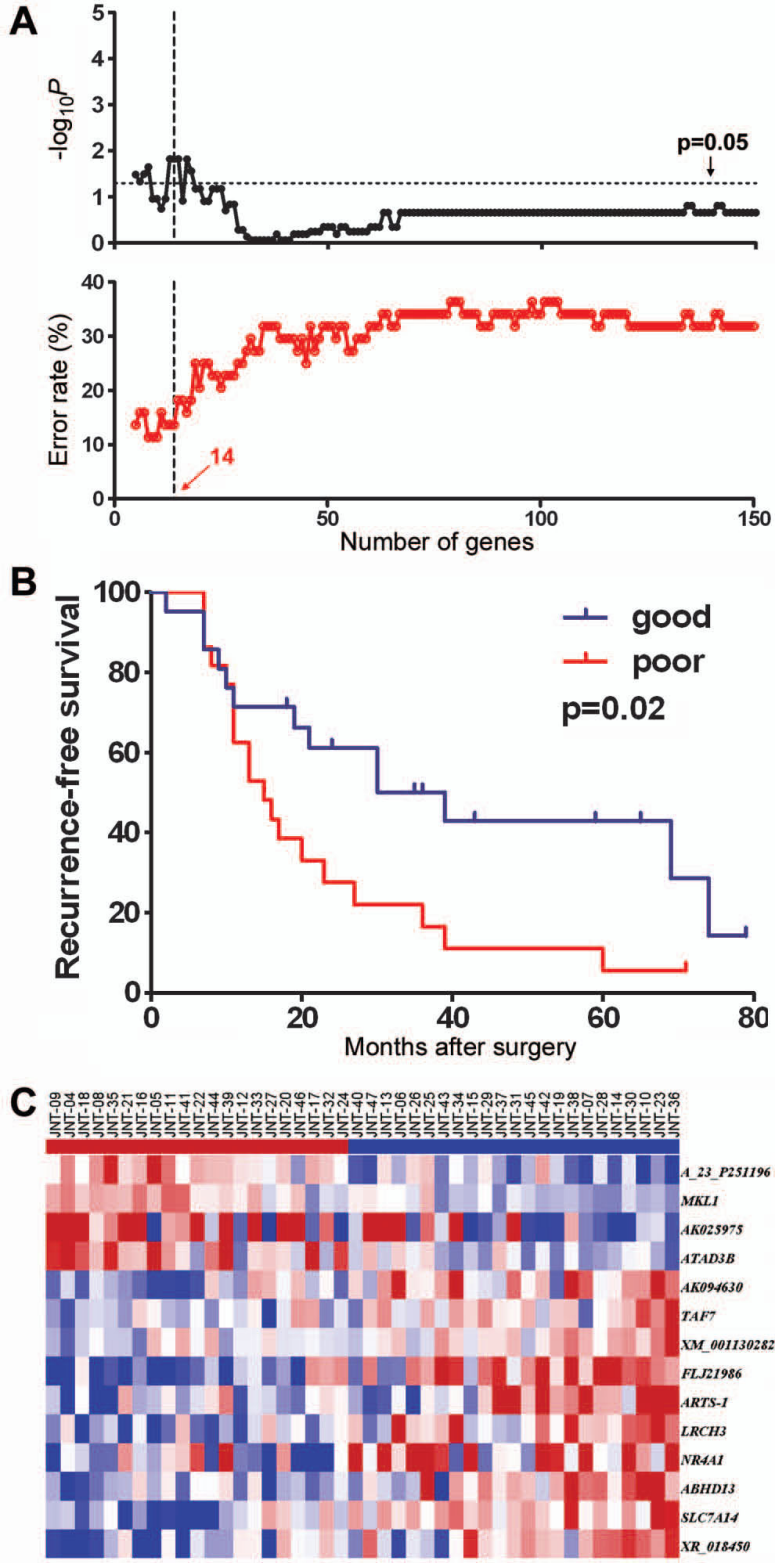
**Recurrence-free survival analysis in the full cohort of HCC patients (N = 44) using gene expression in nontumoral samples**. Expression of 25,073 genes was used as a variable in Cox proportional hazard model. The power of recurrence-free survival prediction for the top-ranked (based on Cox score) genes was assessed using the Nearest Template Prediction (NTP) algorithm and cross-validated with a leave-one-out procedure. (A) Log-rank P-value and error rate of prediction for up to 150 top-ranked genes. (B) Recurrence-free survival curves based of the prediction made using 14 top-ranked genes. (C) Heat map of the 14 gene-based predictors. Red and blue indicate higher or lower than median gene expression, respectively.

Tumor-derived gene expression profiles did not produce a robust classifier of RFS in this cohort. This result led us to consider whether the surrounding liver tissue, not the tumor itself, may yield a molecular predictor associated with HCC recurrence. Indeed, several previous studies have shown that nontumoral tissue profiling in HCC holds great promise with regards to predicting clinical outcomes [11,12,29]. Forty-four nontumoral samples (94% of the entire subject cohort) yielded high-quality gene expression profiles. When both early- and late-recurrence samples were analyzed together, signatures composed of up to 18 top-ranked genes that correlate with disease-free survival were derived (Figure 2). The strongest signature (Figure 2B, p = 0.02 based on prediction during cross-validation) with the fewest number of genes is comprised of 14 genes (Figure 2C).

It has been suggested that distinct gene expression signatures may exist for prediction of early and late recurrence [15]. This is exemplified by the fact that little overlap exists between nontumoral tissue-derived signatures for predicting metastasis-related early recurrence [29], as opposed to those predictive of risk for the late recurrence [12]. Since late recurrence in HCV-infected subjects is likely to be through *de novo *formation of tumors in the diseased liver, we selected a sub-cohort of 28 patents with late (>1 year) recurrence and repeated the cross-validation procedure at both gene and gene set level. Even though a more traditional cut-off for "late" recurrence is 2 years, recent meta-analysis of HCC molecular subclasses suggests that 1 year time point may represent a more biologically-defined cut-off [19].

**Figure 3:**
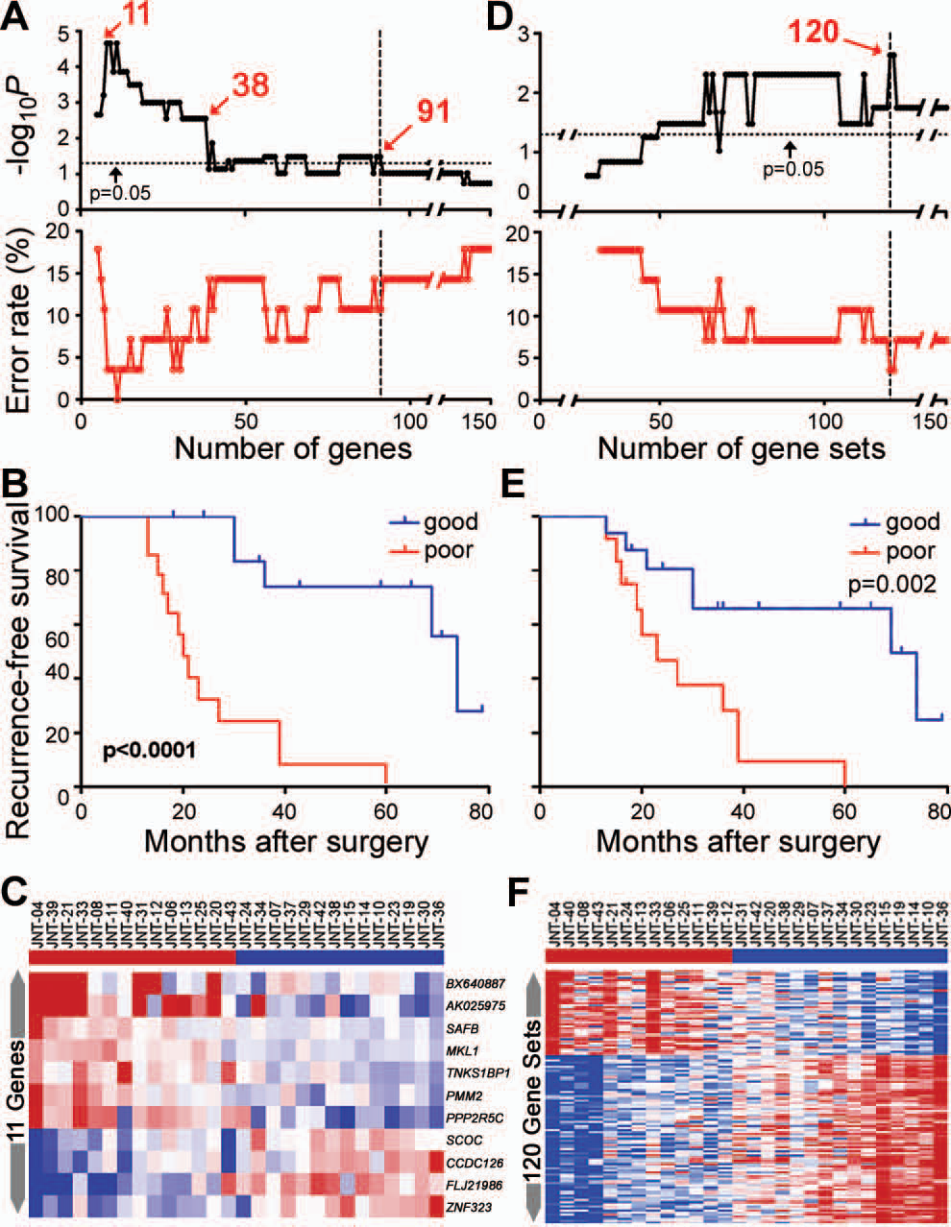
**Recurrence-free survival analysis in HCC patients with late (>1 year) recurrence using gene expression in nontumoral samples**. (A and D) Logrank P-value and error rate of prediction for up to 150 top-ranked genes or gene sets. (B and E) Recurrence-free survival curves based of the prediction made using top-ranked genes (11) or gene sets (120), respectively (see Additional file 1, Tables S5-6 for the lists). (C and F) Heat map of the gene-, or gene set-based predictors. Red and blue indicate higher or lower than median gene expression, respectively.

When individual transcript-level data was used, very strong predictive signatures could be derived for up to 91 genes (Additional file 2, Figure S4; Additional file 1, Table S5). All of the predictors composed of less than 38 genes were highly significant with 11 top-ranked genes producing the strongest signature (Figures 3A-C, p < 0.0001 based on cross-validation, 0% error rate). Importantly, the genes identified as the strongest predictors of RFS that were over expressed in subjects with fastest recurrence have been previously implicated in tumor pathogenesis. For example, megakaryoblastic leukemia factor (MKL)1 has been shown to be required for TGFβ1 stimulation of alpha-smooth muscle actin expression [30], a process which promotes liver fibrosis and cirrhosis. TNKS1BP1 is a tankyrase 1-binding protein and it is known that tankyrase 1 upregulation leads to enhanced access of telomerase to telomeres and promotes clonal expansion of cancer cells [31]. Scaffold attachment factor B (SAFB) is playing a role in transcriptional repression and RNA splicing, and has been shown to be important in numerous cancer-related cellular processes including cell growth, stress response, and apoptosis [32]. Protein phosphatase 2, regulatory subunit B, gamma isoform (PPP2R5C) was shown to function as a tumor suppressor gene. Upon DNA damage, a complex including PPP2R5C and p53 is formed which leads to dephosphorylation of p53, induction of the p53 transcriptional target p21, and the inhibition of cell proliferation [33].

### Multivariable analysis of the nontumoral tissue-derived gene expression signatures of recurrence-free survival in HCV-associated HCC

The poor-prognosis late recurrence signature composed of 11, 38 or 91 top-ranked genes (Figure 3A), along with clinical variables available for the cohort of 28 subjects with recurrence >1 year, was analyzed by univariable Cox-proportional hazard model. Each of the gene expression-based poor-prognosis signatures, tumor diameter and the number of tumors were significantly associated with risk of recurrence in a univariable analysis (Table 3). Next, we examined the signatures in context of the clinical variables that were significant in our study and are also generally accepted as indicative of poor prognosis for recurrence of HCC. Importantly, a multivariable analysis (based on independent prediction of each sample using the cross-validation procedure as detailed above) of each set of top-ranked gene lists combined with two significant clinical variables (number of tumors and tumor diameter) showed that the late recurrence signatures composed of 11 and 38 genes, but not 91 genes, remained significant.

**Table 3:** Association of gene expression poor-prognosis signature and clinical variables with late (>1 year) recurrence from univariate or multivariate analyses.

Variable	Risk ratio	95% CI	P-value
*Univariate Cox proportional hazard model*			
**RFS signature (11 genes)**	**3.13**	**1.75-6.58**	**<0.0001**
**RFS signature (38 genes)**	**2.05**	**1.24-3.54**	**0.005**
**RFS signature (91 genes)**	**1.64**	**1.02-2.71**	**0.04**
**Tumor diameter [cm]**	**2.19**	**1.20-3.95**	**0.01**
**Number of tumors**	**4.28**	**1.24-13.39**	**0.02**
Platelet count [10^4^/μL]	0.91	0.80-1.02	0.11
Portal vein invasion [present]	0.00	0.00-1.93	0.15
Total bilirubin [mg/dL]	1.54	0.37-4.97	0.52
Age	1.02	0.96-1.10	0.53
Alanine aminotransferase [IU/L]	0.99	0.97-1.01	0.55
Indocyanine green [%]	1.02	0.95-1.08	0.56
Prothrombin time [%]	0.99	0.96-1.03	0.64
Liver resection procedure [lobular]	1.16	0.46-2.21	0.71
Fibrosis score	0.93	0.52-1.72	0.80
α-Fetoprotein [ng/μL]	1.00	1.00-1.00	0.85
Tumor differentiation [moderate-poor]	0.91	0.32-3.27	0.87
Sex [Male]	0.93	0.35-2.71	0.89

*Multivariate Cox proportional hazard model*			
**RFS signature (11 genes)**	**3.38**	**1.75-7.77**	**0.0001**
**Number of tumors**	**8.55**	**1.87-46.69**	**0.006**
Tumor diameter [cm]	1.68	0.92-3.19	0.09
			
**RFS signature (38 genes)**	**1.84**	**1.08-3.23**	**0.02**
**Number of tumors**	**4.59**	**1.23-16.11**	**0.02**
**Tumor diameter [cm]**	**2.12**	**1.15-4.03**	**0.02**
			
RFS signature (91 genes)	1.53	0.93-2.60	0.09
Number of tumors	3.58	0.98-12.3	0.05
**Tumor diameter [cm]**	**2.46**	**1.30-4.70**	**0.006**

Cox proportional hazard model analysis was used to assess relative risk for each clinical variable or RFS gene expression signature. Relative risk >1 identifies shortened recurrence-free survival. Significant variables are highlighted in bold font.

### Molecular predictors of tumor recurrence: clinical diagnostic tools or keys to tumor biology?

Identification of gene expression signatures as predictors of the disease outcome has been a very active area of clinical cancer research [15,34]. The gene expression signatures outperform conventional clinicopathologic criteria in high-risk patients and help differentiate treatment options by identifying low-risk individuals [35]. Interestingly, while the signatures usually address the same clinical question, there is only little or no overlap in gene lists derived from different cohorts [15,36]. Most of the individual studies, including our work, are relatively small in size which may limit the potential for direct translation of the molecular signatures to the individual cancer patient management. To address this challenge, large comprehensive meta-analyses integrating both the gene expression and clinicopathologic data from multiple studies have been conducted for breast [35,36], lung [34] and other cancers.

Indeed, a comparison of the applicability of the poor-prognosis signatures from this study to the independent HCC cohort is the ultimate test for the 'biological' validation of the findings. There are two published microarray datasets that profiled nontumoral tissues from HCC subjects as potentially suitable for validation [11,12] which may be used for cross-study validation. Unfortunately, the microarray used by Okamoto et al. [11] lacks comprehensive publicly accessible annotation (Agilent Technologies, personal communications) rendering any meaningful comparison between studies futile. The second study by Hoshida et al. [12] was conducted on formalin-fixed samples and used a specialized microarray containing ~6,000 probes. Not only there is only limited (~20%) overlap in transcripts between the Illumina array (GPL5474, http://www.ncbi.nlm.nih.gov/geo/) and the whole human genome (~44,000 probes) Agilent array used in our work, but also the clinico-pathological variables of the patient cohort used by the authors have not been released to the public (personal communication). Thus, while the cross-study comparisons shall remain as the goal for validation of the findings reported on independent cohorts and array platforms, greater degree of collaboration may be needed in the field of liver tumor biology.

**Figure 4:**
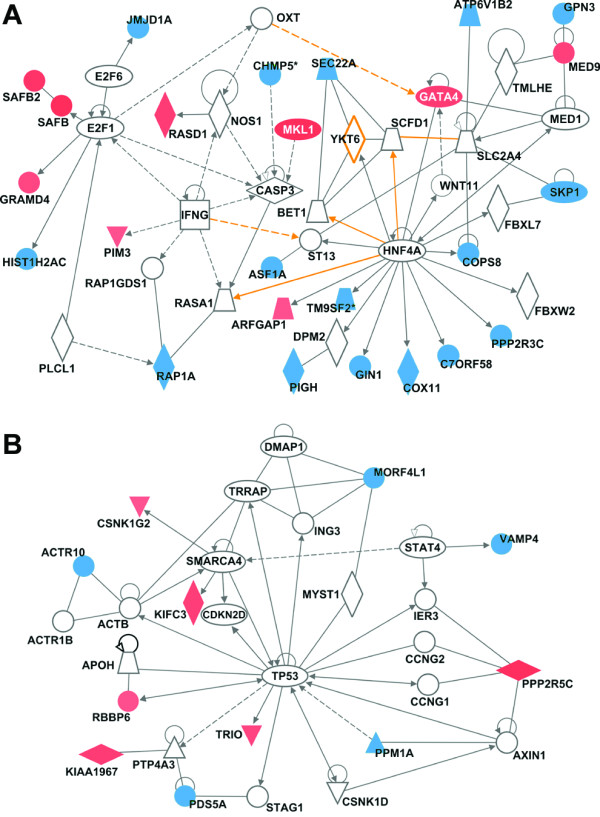
**Molecular networks representing the RFS gene expression signature (91 genes) in HCC patients with late (>1 year) recurrence**. See Additional file 1, Table S7 for a complete list of significant networks. (A) HNF4α- and IFNγ-centered interactomes are top two most significant and interconnected (orange arrows) networks. (B) TP53-centered interactome is the top third significant network. Red and blue colors indicate positive or negative correlation, respectively, of gene expressions to recurrence-free survival based on Cox score. Ellipse, square, triangle, trapezoid, lozenge and circle represent transcription regulator, cytokine, kinase, transporter, enzyme and other molecules, respectively. Red and white represent molecules up-regulated in tumor samples compared to control and molecules incorporated into the network, respectively. Arrows connecting molecules indicate one molecule acts on another and lines indicate one binds to another. Dashed arrows or lines indicate indirect interactions of two molecules.

While the molecular signatures generated in this study may not be clinic-ready without additional meta-analysis once the data becomes publicly available, we posit that late recurrence predictive genes may provide new insights into the oncogenic pathways for HCC development in cirrhotic patients. By examining the biological roles of the predictive genes it may be possible to better understand the process of field cancerization in HCC [37,38] and a transition from cirrhosis to HCC. First, functional pathway analysis was performed using 91 top-ranked genes (Additional file 2, Figure S4; Additional file 1, Table S5) which comprise a comprehensive poor-prognosis signature in nontumoral tissues from subjects with late (>1 year) recurrence (Figure 4 and Additional file 1, Table S7). Interestingly, hepatic nuclear factor (HNF)4α- and interferon (IFN)γ-centered interactomes are top two most significant and interconnected networks (Figure 4A). Expression of HNF4α is an important determinant of HCC progression and the development of aggressive tumor phenotypes in mice [39], while the T-helper 1 inflammatory cells characterized by IFNγ secretion predominate in the liver during chronic HCV infection and are particularly important in disease progression [40]. The TP53-centered interactome is the top third significant network (Figure 4B) and the role of TP53 tumor suppressor pathway is widely recognized as significant contributors to liver carcinogenesis [41].

Second, gene set analysis was used to evaluate the data from the sub-cohort of late (>1 year) recurrence subjects (Figures 3D-F). A number of top-ranked gene sets can serve as predictors (Additional file 1, Table S6) with the profile composed of 120 gene sets being most significant (p = 0.002) with the smallest error rate. Importantly, the gene set-based signature is overabundant in upregulated inflammation- (e.g., nuclear factor-κB, tumor necrosis factor), oxidative stress-, and carcinogenesis- (MYC, MAP kinase) related pathways, and down-regulated cell cycle control-related and protesome pathways. Interestingly, there appears to be great overlap in gene sets associated with poor prognosis outcome between nontumoral tissue and tumors (see Additional file 2, Figure S3, Additional file 1, Table S4) which further supports the theory of field cancerization and progression from cirrhosis to HCC.

## Conclusions

Our study demonstrates that microarray analysis of the nontumoral tissues from subjects with HCV-associated HCC delivers novel molecular signatures of RFS, especially among the late-recurrence subjects. Since late recurrence is likely a *de novo *tumor occurrence in HCV-associated cirrhosis, the biopsy-based gene expression analysis in cirrhotic patients may be a useful method to stratify the risk of tumor occurrence and determine a need for aggressive patient screening. While challenges remain in validating the signatures and translating the findings to clinical practice, we posit that gene expression-based signatures provide important insights into pathobiology of HCC recurrence.

## Methods

### Subjects, specimen collection and study design

Forty-seven subjects with HCV-associated HCC who underwent complete removal of the tumor at the University of Yamanashi Hospital (Yamanashi Prefecture, Japan) between 2000 and 2007 were enrolled in this study. All of the subjects tested positive for HCV and negative for hepatitis B virus. The presence and identification of the hepatitis virus was determined by one or more of the following techniques: (i) presence of anti-HCV and anti-HBV reactive serum proteins, (ii) reverse transcription-PCR for serum HCV-RNA, or (iii) branched DNA-HCV probe assay. Following the tumor resection procedure and recovery after surgery, each subject visited the ambulatory care clinic for additional tests monthly. Serum α-fetoprotein levels were measured every month. In addition, ultrasonography or computed tomography of the liver was performed every 3 and 6 months, respectively. Eight HCV- and HBV-free subjects who were diagnosed with metastatic liver tumors that required surgical resection were enrolled as the control group and nontumoral tissue samples were obtained. All samples were frozen in liquid nitrogen immediately following surgery and kept as -80°C until processed. Informed consent was received from each subject in the study and the study protocol conforms to the ethical guidelines of the 1975 Declaration of Helsinki as reflected in an a priori approval by the Institutional Board on Ethics for Human Science at the University of Yamanashi. All available clinical data for the subjects enrolled in this study are summarized in Table 1. RFS data are provided in Additional file 1, Table S1.

### Statistical analysis

JMP software (ver. 6.0, SAS institute, Cary, NC) was used for statistical analysis of the clinical and molecular (see below) variables. For univariable analysis, two-sample t-test, linear regression, or Logrank test statistical procedures were used to assess what endpoints could be used for predicting prognosis of HCC subjects after surgery. For multivariable analysis, the Cox proportional hazard model was used to calculate hazard ratio and p-value of each parameter. A p value of less than 0.05 was considered significant.

### Microarray analysis

Total RNA was isolated from tumor and nontumoral liver samples (~30 mg) with an RNeasy plus mini kit (Qiagen, Valencia, CA) as detailed by the manufacturer. High quality RNAs was obtained from 43 tumor and 44 nontumoral samples (40 tumor and nontumoral RNA samples were from the same subjects). For microarray analysis, a balanced design was used. Each batch of arrays (Agilent Technologies, Wilmington, DE; whole human genome microarray, cat.#G4112F) was balanced with regards to the tumor stage and RFS and contained both inter-batch and intra-batch replicates.

RNA samples were hybridized to arrays individually; none were pooled. RNA amplifications and labeling were performed using Low RNA Input Linear Amplification kits (Agilent Technologies). For hybridization, 1.2 μg of total RNA from each sample, or human reference RNA (Stratagene, La Jolla, CA; Cat#740000) was amplified and labeled with fluorescent dye (Cy5 and Cy3, respectively). RNA labeling, microarray hybridization, washing and scanning were performed according to the manufacturer's instructions. Feature extraction, normalization of the raw data and data filtering were performed using the Agilent Feature Extraction software (v8.5, Agilent Technologies). Raw microarray data was archived in Gene Expression Omnibus (GSE17856) and is available to the public. The log_2 _ratio of Cy5/Cy3 intensity was normalized using LOWESS smoothing to eliminate intensity bias of features. Transcripts with fewer than 70% available data across samples were excluded from the analysis, reducing the probe list to 25,073 transcripts. Available data are defined as those probes that are neither saturated nor below the limit of quantification. Inter-batch normalization was carried out using Distance Weighted Discrimination procedure [42].

### Biological pathway analysis on tumor samples

Differentially expressed genes between control and tumor sample groups were identified by using Significant Analysis of Microarray analysis [43]. Individual genes with false discovery rate (FDR) = 0 in the univariable test under 1,000 random permutations were considered significant. Selected genes were analyzed using Ingenuity Pathways Analysis (v. 7.1; Ingenuity Systems, Redwood City, CA) to determine canonical pathways that are enriched among the significant transcripts. SAFEGUI [44] was used to compare gene expression level between HCC tumor samples and control nontumoral liver samples from subjects with metastatic liver tumors based on Gene Ontology (GO) biological process categories, or Kyoto Encyclopedia of Genes and Genomes (KEGG) pathways. P-values less than 0.01 were considered to be significant.

### Gene expression-based recurrence-free survival (RFS) predictor

Tumor and adjacent nontumoral liver tissues were considered separately to define an RFS-predictive signature on each type of tissue. In each dataset, genes whose expression was associated with time to recurrence were selected by means of the high absolute Cox score and prediction analysis was performed by evaluating the expression status of the signature using the nearest template prediction method as described elsewhere[12] and implemented in GenePattern software (ver.3.1.1, http://www.broad.mit.edu/cancer/software/genepattern/). The signatures were validated using a leave-one-out cross-validation prediction procedure. In brief, each sample was left out one-by-one and a disease-free survival-correlated signature was deduced using remaining samples (selecting marker genes based on top-ranked highest absolute Cox score). A predicted class label was assigned to the left-out sample based on the closest "template" using the nearest template prediction algorithm. A maximum of 200 top-ranked genes were used to make predictions in each analysis. The outcome of cross-validation based prediction was assessed by Logrank test and the "error rate," defined here as the ratio of the number of subjects predicted incorrectly to that in the model. In parallel, the "gene set" analysis (GSA) was also used to determine the significance of pre-defined sets of genes with respect to an outcome variable, such as a RFS time. GSA package for R http://www-stat.stanford.edu/~tibs/GSA/ was utilized. The output from GSA was used to generate RFS predictors in the same manner as detailed above for gene-level data and validated using a leave-one-out cross-validation procedure.

## List of Abbreviations

HCC: Hepatocellular Carcinoma; HCV: Hepatitis C Virus; FDR: False Discovery Rate; GO: Gene Ontology; KEGG: Kyoto Encyclopedia of Genes and Genomes; RFS: Recurrence-Free Survival; SAFE: Significance Analysis of Function and Expression.

## Competing interests

The authors declare that they have no competing interests.

## Authors' contributions

MT participated in the design of the study, carried out the molecular and statistical analyses and drafted the manuscript; JP carried out the statistical analyses and participated in writing of the manuscript; HK, MM and HF designed the study, performed sample analyses and assisted with the development of the manuscript; IR designed the study, participated in the analyses of the data and was responsible for writing of the manuscript. All authors read and approved the final manuscript.

## Supplementary Material

Additional file 1 table S1. Clinical outcomes for each individual patient included in this study. table S2. Molecular networks (Ingenuity^® ^pathways analysis) differentially modulated between control (nontumoral samples from patients with metastatic liver tumors) tissue and HCC tumors. table S3. GO categories and KEGG pathways that differ significantly between control and HCC tumor samples were identified using SAFE analysis (see Methods). table S4. Sixty seven top-ranked gene sets used for recurrence prediction analysis in tumor samples (see Figure 3C for the heat map). table S5. Ninety one top-ranked genes used for recurrence prediction analysis in nontumoral samples from late (>1 year) recurrence subjects (see Figure 3C for the heat map). table S6. One hundred twenty top-ranked gene sets used for recurrence prediction analysis in nontumoral samples from late (>1 year) recurrence subjects (see Figure 3F for the heat map). table S7. Molecular networks representing the recurrence-free survival gene expression signature (91 genes) in HCC patients with late (>1 year) recurrence. Click here for file

Additional file 2 Figure S1. TGFβ1- and MYC-centered interactomes are most significantly up-regulated molecular networks in HCC tumors (see Additional file 1, Table S2-3 for a full list of the significant pathways). Ellipse, square, triangle, trapezoid, lozenge and circle represent transcription regulator, cytokine, kinase, transporter, enzyme and other molecules, respectively. Red and white represent molecules up-regulated in tumor samples compared to control and molecules incorporated into the network, respectively. Arrows connecting molecules indicate one molecule acts on another and lines indicate one binds to another. Dashed arrows or lines indicate indirect interactions of two molecules. Figure S2. Analysis of the dissimilarity (Spearman's correlation) between gene expression profiles of the nontumoral (top) or tumoral (bottom) samples used in this study. Expression of 25,073 genes was used as a variable to create the heat map of each dissimilarity matrix. The color bar represents the degree of dissimilarity with red exhibiting high degree of dissimilarity and blue - high similarity. Figure S3. Recurrence-free survival analysis in HCC patients using gene set analysis in tumor samples. (A) Logrank P-value and error rate of prediction for up to 150 top-ranked gene sets. (B) Recurrence-free survival curves (Kaplan-Meier method) based of the prediction made using 67 top-ranked gene sets (Additional file 1, Table S4). Red and blue represent the subjects predicted to have poor or good recurrence-free survival, respectively. (C) Heatmap of the 67 gene set-based survival signature from tumor samples. Red and blue indicate high or low gene set score, respectively. Subjects (x-axis) are sorted by means of cosine distance to 'poor' template, and gene sets (y-axis) are sorted by Cox score (Additional file 1, Table S4). Figure S4. Recurrence-free survival analysis in HCC patients with late (>1 year) recurrence using gene expression (91 genes) in nontumoral samples. (A) 91 genes selected based on log-rank P-value and error rate of prediction were used to construct recurrence-free survival curves (B). (C) Heat map of the gene-based survival signature with 91 genes. Click here for file
