# Supplementary material for: Gene expression in nontumoral liver tissue and recurrence-free survival in hepatitis C virus-positive hepatocellular carcinoma
Source: Mol Cancer. 2010 Apr 9;9:74. doi: 10.1186/1476-4598-9-74 (PMC2856554; doi:10.1186/1476-4598-9-74)
Supplement: Additional file 1 — table S1. Clinical outcomes for each individual patient included in this study. table S2. Molecular networks (Ingenuity® pathways analysis) differentially modulated between control (nontumoral samples from patients with metastatic liver tumors) tissue and HCC tumors. table S3. GO categories and KEGG pathways that differ significantly between control and HCC tumor samples were identified using SAFE analysis (see Methods). table S4. Sixty seven top-ranked gene sets used for recurrence prediction analysis in tumor samples (see Figure 3C for the heat map). table S5. Ninety one top-ranked genes used for recurrence prediction analysis in nontumoral samples from late (>1 year) recurrence subjects (see Figure 3C for the heat map). table S6. One hundred twenty top-ranked gene sets used for recurrence prediction analysis in nontumoral samples from late (>1 year) recurrence subjects (see Figure 3F for the heat map). table S7. Molecular networks representing the recurrence-free survival gene expression signature (91 genes) in HCC patients with late (>1 year) recurrence. [file 1476-4598-9-74-S1.DOC]

**Table S1.** Clinical outcomes for each individual patient included in this study.

| Experiment ID | Tumor | Non-tumor | RFS | Censored=1 |
| --- | --- | --- | --- | --- |
|  | (X, when available) | | (month) |  |
| J(N)T-01 | X |  | 15 | 0 |
| J(N)T-04 | X | X | 15 | 0 |
| J(N)T-05 |  | X | 10 | 0 |
| J(N)T-06 | X | X | 39 | 0 |
| J(N)T-07 | X | X | 74 | 0 |
| J(N)T-08 |  | X | 23 | 0 |
| J(N)T-09 |  | X | 0 | 0 |
| J(N)T-10 |  | X | 79 | 1 |
| J(N)T-11 | X | X | 20 | 0 |
| J(N)T-12 | X | X | 27 | 0 |
| J(N)T-13 | X | X | 60 | 0 |
| J(N)T-14 | X | X | 69 | 0 |
| J(N)T-15 | X | X | 71 | 1 |
| J(N)T-16 | X | X | 7 | 0 |
| J(N)T-17 | X | X | 10 | 0 |
| J(N)T-18 | X | X | 7 | 0 |
| J(N)T-19 | X | X | 65 | 1 |
| J(N)T-20 | X | X | 17 | 0 |
| J(N)T-21 | X | X | 16 | 0 |
| J(N)T-22 | X | X | 8 | 0 |
| J(N)T-23 | X | X | 59 | 1 |
| J(N)T-24 | X | X | 36 | 0 |
| J(N)T-25 | X | X | 39 | 0 |
| J(N)T-26 | X | X | 11 | 0 |
| J(N)T-27 | X | X | 2 | 0 |
| J(N)T-28 | X | X | 9 | 0 |
| J(N)T-29 | X | X | 30 | 0 |
| J(N)T-30 | X | X | 43 | 1 |
| J(N)T-31 | X | X | 21 | 0 |
| J(N)T-32 | X | X | 11 | 0 |
| J(N)T-33 | X | X | 13 | 0 |
| J(N)T-34 | X | X | 30 | 0 |
| J(N)T-35 | X | X | 11 | 0 |
| J(N)T-36 | X | X | 36 | 1 |
| J(N)T-37 | X | X | 35 | 1 |
| J(N)T-38 | X | X | 24 | 1 |
| J(N)T-39 | X | X | 13 | 0 |
| J(N)T-40 | X | X | 19 | 0 |
| J(N)T-41 | X | X | 7 | 0 |
| J(N)T-42 | X | X | 18 | 1 |
| J(N)T-43 | X | X | 17 | 1 |
| J(N)T-44 | X | X | 11 | 0 |
| J(N)T-45 | X | X | 7 | 0 |
| J(N)T-46 | X | X | 9 | 1 |
| J(N)T-47 | X | X | 7 | 0 |
| J(N)T-48 | X |  | 16 | 0 |
| J(N)T-49 | X |  | 11 | 0 |
| JC-01 |  | X |  |  |
| JC-02 |  | X |  |  |
| JC-03 |  | X |  |  |
| JC-06 |  | X |  |  |
| JC-07 |  | X |  |  |
| JC-09 |  | X |  |  |
| JC-10 |  | X |  |  |
| JC-11 |  | X |  |  |

**Table S2.** Molecular networks (Ingenuity® pathways analysis) differentially modulated between control (nontumoral samples from patients with metastatic liver tumors) tissue and HCC tumors.

| Rank | Score* | Focus molecules | Molecular functions |
| --- | --- | --- | --- |
| **Up-regulated** | | | |
| 1 | 21 | 14 | Cell Cycle, Cancer, Tumor Morphology |
| 2 | 5 | 4 | Cell Death, Hematological Disease, Immunological Disease |
| **Down-regulated** | | |  |
| 1 | 45 | 35 | Organismal Injury and Abnormalities, Hematological System Development and Function, Tissue Development |
| 2 | 11 | 14 | Cancer, Cell Death, Hematological Disease |
| 3 | 10 | 15 | Cell Cycle, Cancer, DNA Replication, Recombination, and Repair |
| 4 | 9 | 14 | Antigen Presentation, Cell-To-Cell Signaling and Interaction, Hematological System Development and Function |
| 5 | 9 | 14 | Cell-To-Cell Signaling and Interaction, Tissue Development, Cellular Movement |
| 6 | 9 | 14 | Cellular Movement, Cell Death, Cellular Growth and Proliferation |
| 7 | 9 | 14 | Cellular Growth and Proliferation, Cellular Development, Cancer |
| 8 | 8 | 12 | Metabolic Disease, Antigen Presentation, Cell-mediated Immune Response |
| 9 | 8 | 13 | Cellular Growth and Proliferation, Cell Death, Cancer |
| 10 | 8 | 13 | Gene Expression, Cell Death, Cell Cycle |
| 11 | 8 | 13 | Cellular Growth and Proliferation, Cell Death, Cellular Development |
| 12 | 7 | 12 | Cellular Growth and Proliferation, Endocrine System Development and Function, Cell Death |

Genes whose expression was significantly different between control liver and tumor tissue (FDR=0) were selected as detailed in Methods. Up-regulated and down-regulated genes were analyzed independently.

*Ingenuity-derived significance score for the molecular network.

**Table S3.** GO categories and KEGG pathways that differ significantly between control and HCC tumor samples were identified using SAFE analysis (see Methods).

| category | Size | P-value | Term |
| --- | --- | --- | --- |
| ***Go category*** |  |  |  |
| GO:0032102 | 10 | 0.002 | negative regulation of response to external stimulus |
| GO:0043691 | 7 | 0.002 | reverse cholesterol transport |
| GO:0050728 | 9 | 0.002 | negative regulation of inflammatory response |
| GO:0031348 | 12 | 0.003 | negative regulation of defense response |
| GO:0050727 | 31 | 0.003 | regulation of inflammatory response |
| GO:0050885 | 21 | 0.003 | neuromuscular process controlling balance |
| GO:0031960 | 16 | 0.004 | response to corticosteroid stimulus |
| GO:0042130 | 8 | 0.004 | negative regulation of T cell proliferation |
| GO:0051384 | 15 | 0.004 | response to glucocorticoid stimulus |
| GO:0010038 | 52 | 0.005 | response to metal ion |
| GO:0015918 | 20 | 0.006 | sterol transport |
| GO:0030301 | 20 | 0.006 | cholesterol transport |
| GO:0032945 | 11 | 0.006 | negative regulation of mononuclear cell proliferation |
| GO:0045471 | 11 | 0.006 | response to ethanol |
| GO:0050672 | 11 | 0.006 | negative regulation of lymphocyte proliferation |
| GO:0050848 | 8 | 0.006 | regulation of calcium-mediated signaling |
| GO:0051241 | 55 | 0.006 | negative regulation of multicellular organismal process |
| GO:0001933 | 15 | 0.007 | negative regulation of protein amino acid phosphorylation |
| GO:0002455 | 34 | 0.007 | humoral immune response mediated by circulating immunoglobulin |
| GO:0006957 | 12 | 0.007 | complement activation, alternative pathway |
| GO:0022600 | 18 | 0.007 | digestive system process |
| GO:0032094 | 5 | 0.007 | response to food |
| GO:0006970 | 10 | 0.008 | response to osmotic stress |
| GO:0007611 | 40 | 0.008 | learning and/or memory |
| GO:0008206 | 14 | 0.008 | bile acid metabolic process |
| GO:0009086 | 12 | 0.008 | methionine biosynthetic process |
| GO:0042445 | 80 | 0.008 | hormone metabolic process |
| GO:0045986 | 5 | 0.008 | negative regulation of smooth muscle contraction |
| GO:0003084 | 5 | 0.009 | positive regulation of systemic arterial blood pressure |
| GO:0006904 | 26 | 0.009 | vesicle docking during exocytosis |
| GO:0030195 | 18 | 0.009 | negative regulation of blood coagulation |
| GO:0046329 | 6 | 0.009 | negative regulation of JNK cascade |
| GO:0002541 | 38 | 0.01 | activation of plasma proteins during acute inflammatory response |
| GO:0003044 | 10 | 0.01 | regulation of systemic arterial blood pressure mediated by a chemical signal |
| GO:0006546 | 9 | 0.01 | glycine catabolic process |
| GO:0006613 | 29 | 0.01 | cotranslational protein targeting to membrane |
| GO:0006956 | 38 | 0.01 | complement activation |
| GO:0009617 | 47 | 0.01 | response to bacterium |
| GO:0031400 | 22 | 0.01 | negative regulation of protein modification process |
| GO:0043496 | 9 | 0.01 | regulation of protein homodimerization activity |
| ***KEGG pathway*** | | |  |
| KEGG:00260 | 54 | 0.002 | Glycine, serine and threonine metabolism |
| KEGG:00360 | 27 | 0.005 | Phenylalanine metabolism |
| KEGG:03022 | 40 | 0.008 | Basal transcription factors |
| KEGG:00361 | 16 | 0.01 | gamma-Hexachlorocyclohexane degradation |
| KEGG:00330 | 39 | 0.02 | Arginine and proline metabolism |
| KEGG:00401 | 6 | 0.02 | Novobiocin biosynthesis |
| KEGG:00903 | 29 | 0.021 | Limonene and pinene degradation |
| KEGG:04610 | 75 | 0.022 | Complement and coagulation cascades |
| KEGG:04350 | 121 | 0.024 | TGF-beta signaling pathway |
| KEGG:00363 | 6 | 0.025 | Bisphenol A degradation |
| KEGG:04620 | 91 | 0.026 | Toll-like receptor signaling pathway |
| KEGG:00860 | 41 | 0.031 | Porphyrin and chlorophyll metabolism |
| KEGG:00830 | 54 | 0.035 | Retinol metabolism |
| KEGG:00982 | 69 | 0.041 | Drug metabolism - cytochrome P450 |
| KEGG:00980 | 66 | 0.042 | Metabolism of xenobiotics by cytochrome P450 |
| KEGG:00591 | 23 | 0.043 | Linoleic acid metabolism |
| KEGG:00232 | 6 | 0.045 | Caffeine metabolism |
| KEGG:00600 | 47 | 0.045 | Sphingolipid metabolism |
| KEGG:04115 | 99 | 0.047 | p53 signaling pathway |
| KEGG:04614 | 14 | 0.047 | Renin-angiotensin system |
| KEGG:00310 | 59 | 0.049 | Lysine degradation |

**Table S4.** Sixty seven top-ranked gene sets used for recurrence prediction analysis in tumor samples (see Figure 3C for the heat map).

| Gene set | Cox score |
| --- | --- |
| MTORPATHWAY | 2.39 |
| HDACI_COLON_TSABUT_UP | 2.38 |
| GENOTOXINS_4HRS_DISCR | 2.35 |
| HDACI_COLON_BUT16HRS_UP | 2.34 |
| TGFBETA_C1_UP | 2.27 |
| CMV_8HRS_DN | 2.27 |
| HDACI_COLON_BUT12HRS_UP | 2.27 |
| HDACI_COLON_TSA2HRS_UP | 2.26 |
| HDACI_COLON_BUT_UP | 2.26 |
| HDACI_COLON_TSA_UP | 2.26 |
| UV_ESR_OLD_UNREG | 2.24 |
| HDACI_COLON_BUT48HRS_UP | 2.21 |
| TGFBETA_EARLY_UP | 2.21 |
| MENSE_HYPOXIA_UP | 2.20 |
| UVC_TTD_4HR_UP | 2.20 |
| UVC_TTD_ALL_UP | 2.19 |
| TGFBETA_ALL_UP | 2.19 |
| TSADAC_RKOSILENT_UP | 2.18 |
| HYPOXIA_RCC_UP | 2.18 |
| CMV_HCMV_TIMECOURSE_6HRS_DN | 2.17 |
| CALRES_RHESUS_UP | 2.17 |
| YAO_P4_KO_VS_WT_DN | 2.16 |
| UVC_LOW_C3_DN | 2.15 |
| ZHAN_MM_CD138_MS_VS_REST | 2.14 |
| GUO_HEX_UP | 2.13 |
| HYPERTROPHY_MODEL | 2.13 |
| HDACI_COLON_BUT24HRS_UP | 2.11 |
| UVC_TTD-XPCS_COMMON_UP | 2.09 |
| CORDERO_KRAS_KD_VS_CONTROL_UP | 2.09 |
| ALZHEIMERS_INCIPIENT_UP | 2.08 |
| FLECHNER_KIDNEY_TRANSPLANT_WELL_PBL_DN | 2.07 |
| UVC_XPCS_ALL_UP | 2.07 |
| HDACI_COLON_TSA48HRS_UP | 2.05 |
| AGED_RHESUS_DN | 2.05 |
| ADIPOGENESIS_HMSC_CLASS1_UP | 2.05 |
| NGUYEN_KERATO_DN | 2.04 |
| HSA04150_MTOR_SIGNALING_PATHWAY | 2.04 |
| UVB_NHEK3_ALL | 2.04 |
| SHEPARD_POS_REG_OF_CELL_PROLIFERATION | 2.04 |
| TUMOR_SUPRESSOR | 2.04 |
| FERRANDO_MLL_T_ALL_UP | 2.03 |
| TGFBETA_LATE_UP | 2.03 |
| TAKEDA_NUP8_HOXA9_6H_DN | 2.02 |
| POD1_KO_MOST_DN | 2.02 |
| UVC_XPCS_8HR_UP | 2.02 |
| H2O2_CSBRESCUED_UP | 2.01 |
| HIPPOCAMPUS_DEVELOPMENT_NEONATAL | 2.01 |
| UVB_NHEK3_C5 | 2.01 |
| ST_DIFFERENTIATION_PATHWAY_IN_PC12_CELLS | 2.01 |
| INTEGRIN_MEDIATED_CELL_ADHESION_KEGG | 2.01 |
| YAGI_AML_PROG_ASSOC | 2.00 |
| BRCA1_SW480_DN | 2.00 |
| CMV-UV_HCMV_6HRS_DN | 1.99 |
| METPATHWAY | 1.99 |
| HSA04360_AXON_GUIDANCE | 1.99 |
| HSA05217_BASAL_CELL_CARCINOMA | 1.99 |
| WNT_SIGNALING | 1.99 |
| HSC_LATEPROGENITORS_ADULT | -1.99 |
| AGED_MOUSE_NEOCORTEX_DN | -2.00 |
| HSC_LATEPROGENITORS_SHARED | -2.00 |
| HSC_LATEPROGENITORS_FETAL | -2.00 |
| HSA00760_NICOTINATE_AND_NICOTINAMIDE_METABOLISM | -2.04 |
| HSA05214_GLIOMA | -2.06 |
| ZHAN_MM_MOLECULAR_CLASSI_DN | -2.09 |
| XU_CBP_DN | -2.16 |
| HSA05223_NON_SMALL_CELL_LUNG_CANCER | -2.27 |
| HSA00740_RIBOFLAVIN_METABOLISM | -2.32 |

**Table S5.** Ninety one top-ranked genes used for recurrence prediction analysis in nontumoral samples from late (>1 year) recurrence subjects (see Figure 4C for the heat map).

| Agilent probe | Primary accession | Gene Symbol | Gene name | Cox score |
| --- | --- | --- | --- | --- |
| A_24_P694738 | BX640887 | LOC388796 | hypothetical LOC388796 | 3.68 |
| A_32_P406142 | AK025975 |  |  | 3.53 |
| A_23_P208446 | NM_002967 | SAFB | scaffold attachment factor B | 3.12 |
| A_23_P348138 | NM_020831 | MKL1 | megakaryoblastic leukemia (translocation) 1 | 3.12 |
| A_23_P418597 | NM_033396 | TNKS1BP1 | tankyrase 1 binding protein 1, 182kDa | 3.11 |
| A_23_P432360 | NM_000303 | PMM2 | phosphomannomutase 2 | 3.09 |
| A_24_P76898 | AL834350 | PPP2R5C | protein phosphatase 2, regulatory subunit B', gamma isoform | 3.07 |
| A_23_P27894 | NM_014649 | SAFB2 | scaffold attachment factor B2 | 2.99 |
| A_23_P384761 | NM_002052 | GATA4 | GATA binding protein 4 | 2.85 |
| A_23_P118392 | NM_016084 | RASD1 | RAS, dexamethasone-induced 1 | 2.83 |
| A_23_P31686 | NM_021174 | KIAA1967 | KIAA1967 | 2.82 |
| A_23_P74688 | NM_031921 | ATAD3B | ATPase family, AAA domain containing 3B | 2.79 |
| A_23_P54576 | NM_005550 | KIFC3 | kinesin family member C3 | 2.78 |
| A_23_P410507 | NM_004158 | PSPN | persephin | 2.77 |
| A_23_P66402 | NM_018019 | MED9 | mediator of RNA polymerase II transcription, subunit 9 homolog (S. cerevisiae) | 2.76 |
| A_24_P383450 | NM_203434 | IER5L | immediate early response 5-like | 2.70 |
| A_32_P168853 | AK123481 |  |  | 2.69 |
| A_23_P425880 | NM_007118 | TRIO | triple functional domain (PTPRF interacting) | 2.68 |
| A_24_P23258 | NM_015124 | DIP | death-inducing-protein | 2.67 |
| A_32_P139909 | CD607715 |  |  | 2.61 |
| A_24_P131785 | NM_003704 | C4orf8 | chromosome 4 open reading frame 8 | 2.60 |
| A_23_P251196 |  |  |  | 2.58 |
| A_23_P157247 | NM_152689 | MGC9712 | hypothetical protein MGC9712 | 2.52 |
| A_32_P151152 | AA451708 |  |  | 2.51 |
| A_23_P363206 | NM_006910 | RBBP6 | retinoblastoma binding protein 6 | 2.51 |
| A_23_P337550 | NM_017602 | OTUD5 | OTU domain containing 5 | 2.50 |
| A_23_P210379 | NM_175609 | ARFGAP1 | ADP-ribosylation factor GTPase activating protein 1 | 2.50 |
| A_23_P61398 | NM_001001852 | PIM3 | pim-3 oncogene | 2.49 |
| A_23_P421175 | NM_198488 | FAM83H | family with sequence similarity 83, member H | 2.48 |
| A_23_P56140 | NM_001319 | CSNK1G2 | casein kinase 1, gamma 2 | 2.46 |
| A_23_P123018 | NM_001039145 | WBSCR23 | Williams-Beuren syndrome chromosome region 23 | 2.46 |
| A_32_P85539 |  |  |  | -2.43 |
| A_24_P398691 | NM_032859 | ABHD13 | abhydrolase domain containing 13 | -2.44 |
| A_32_P2342 | BX641010 |  |  | -2.44 |
| A_23_P395075 | NM_018433 | JMJD1A | jumonji domain containing 1A | -2.44 |
| A_23_P372860 | NM_003512 | HIST1H2AC | histone cluster 1, H2ac | -2.44 |
| A_23_P57304 | NM_006134 | TMEM50B | transmembrane protein 50B | -2.44 |
| A_23_P140154 | AL080118 | C14orf109 | chromosome 14 open reading frame 109 | -2.45 |
| A_23_P51754 | NM_001010935 | RAP1A | RAP1A, member of RAS oncogene family | -2.45 |
| A_23_P116999 | NM_016301 | ATPBD1C | ATP binding domain 1 family, member C | -2.45 |
| A_32_P164314 | NM_206839 | MORF4L1 | mortality factor 4 like 1 | -2.45 |
| A_23_P2884 | NM_004569 | PIGH | phosphatidylinositol glycan anchor biosynthesis, class H | -2.45 |
| A_24_P767901 | XR_018450 | LOC648960 | hypothetical protein LOC648960 | -2.45 |
| A_24_P118874 |  |  |  | -2.46 |
| A_24_P143843 | XM_001130282 |  |  | -2.46 |
| A_32_P429872 | NM_016097 | IER3IP1 | immediate early response 3 interacting protein 1 | -2.46 |
| A_23_P251965 | BC008386 | ZNF273 | zinc finger protein 273 | -2.46 |
| A_32_P29140 | AA344632 |  |  | -2.47 |
| A_24_P272653 |  |  |  | -2.49 |
| A_23_P256903 | AF086546 |  |  | -2.49 |
| A_24_P870463 | AL137389 |  |  | -2.49 |
| A_23_P112260 | NM_004125 | LOC552891 | hypothetical protein LOC552891 | -2.50 |
| A_24_P186216 | BC041361 | SCC-112 | SCC-112 protein | -2.51 |
| A_24_P112087 | NM_014034 | ASF1A | ASF1 anti-silencing function 1 homolog A (S. cerevisiae) | -2.51 |
| A_24_P942030 | AK056124 | VAMP4 | vesicle-associated membrane protein 4 | -2.52 |
| A_23_P31844 | NM_001693 | ATP6V1B2 | ATPase, H+ transporting, lysosomal 56/58kDa, V1 subunit B2 | -2.52 |
| A_32_P24531 | BC014023 |  |  | -2.53 |
| A_23_P133432 | NM_170679 | SKP1A | S-phase kinase-associated protein 1A (p19A) | -2.53 |
| A_24_P186862 | NM_016410 | CHMP5 | chromatin modifying protein 5 | -2.53 |
| A_24_P44341 | NM_020121 | UGCGL2 | UDP-glucose ceramide glucosyltransferase-like 2 | -2.54 |
| A_23_P151679 | NM_021003 | PPM1A | protein phosphatase 1A (formerly 2C), magnesium-dependent, alpha isoform | -2.55 |
| A_23_P92824 | NM_031482 | ATG10 | ATG10 autophagy related 10 homolog (S. cerevisiae) | -2.55 |
| A_24_P210399 | NM_004800 | TM9SF2 | transmembrane 9 superfamily member 2 | -2.56 |
| A_23_P151394 | NM_004800 | TM9SF2 | transmembrane 9 superfamily member 2 | -2.56 |
| A_23_P360245 | NM_145266 | NUDCD2 | NudC domain containing 2 | -2.57 |
| A_23_P144257 | NM_198189 | COPS8 | COP9 constitutive photomorphogenic homolog subunit 8 (Arabidopsis) | -2.57 |
| A_32_P233019 | NM_012430 | SEC22A | SEC22 vesicle trafficking protein homolog A (S. cerevisiae) | -2.59 |
| A_23_P88201 | NM_017917 | PPP2R3C | protein phosphatase 2 (formerly 2A), regulatory subunit B'', gamma | -2.59 |
| A_23_P206526 |  |  |  | -2.59 |
| A_23_P99579 | NM_032490 | C14orf142 | chromosome 14 open reading frame 142 | -2.59 |
| A_32_P128496 | DQ786272 |  |  | -2.62 |
| A_23_P165788 | NM_001008489 | PHOSPHO2 | phosphatase, orphan 2 | -2.64 |
| A_32_P125135 | AL834140 |  |  | -2.64 |
| A_23_P168587 | NM_138771 | CCDC126 | coiled-coil domain containing 126 | -2.65 |
| A_23_P149695 | BC098428 | C1orf203 | chromosome 1 open reading frame 203 | -2.65 |
| A_23_P168592 | NM_138771 | CCDC126 | coiled-coil domain containing 126 | -2.65 |
| A_24_P278156 | NM_018112 | TMEM38B | transmembrane protein 38B | -2.67 |
| A_23_P110611 | NM_017676 | ZH2C2 | zinc finger, H2C2 domain containing | -2.68 |
| A_24_P351435 | NM_016302 | CRBN | cereblon | -2.69 |
| A_23_P80122 | NM_004627 | WRB | tryptophan rich basic protein | -2.69 |
| A_23_P83278 | NM_016410 | CHMP5 | chromatin modifying protein 5 | -2.70 |
| A_24_P180363 | AK022059 |  |  | -2.74 |
| A_32_P82424 | XR_019210 | LOC647252 | similar to Charged multivesicular body protein 5 (Chromatin-modifying protein 5) (Vacuolar protein sorting 60) (Vps60) (hVps60) (SNF7 domain-containing protein 2) | -2.76 |
| A_24_P324506 |  |  |  | -2.84 |
| A_23_P200252 | NM_032324 | C1orf57 | chromosome 1 open reading frame 57 | -2.89 |
| A_24_P73943 | NM_004375 | COX11 | COX11 homolog, cytochrome c oxidase assembly protein (yeast) | -2.91 |
| A_23_P151690 | NM_018477 | ACTR10 | actin-related protein 10 homolog (S. cerevisiae) | -2.99 |
| A_23_P167293 | NM_032547 | SCOC | short coiled-coil protein | -3.08 |
| A_24_P354954 | NM_138771 | CCDC126 | coiled-coil domain containing 126 | -3.09 |
| A_24_P187799 | NM_024913 | FLJ21986 | hypothetical protein FLJ21986 | -3.20 |
| A_24_P137713 | NM_030899 | ZNF323 | zinc finger protein 323 | -3.24 |

**Table S6.** One hundred twenty top-ranked gene sets used for recurrence prediction analysis in nontumoral samples from late (>1 year) recurrence subjects (see Figure 4F for the heat map).

| Gene set | Cox score |
| --- | --- |
| NFKBPATHWAY | 2.34 |
| LIN_WNT_UP | 2.22 |
| KERATINOCYTEPATHWAY | 2.21 |
| TIDPATHWAY | 2.20 |
| FALT_BCLL_UP | 2.19 |
| TNFR2PATHWAY | 2.17 |
| GLUTAMATE_METABOLISM | 2.17 |
| MAPKPATHWAY | 2.14 |
| AGUIRRE_PANCREAS_CHR19 | 2.13 |
| HEARTFAILURE_ATRIA_UP | 2.13 |
| LIAN_MYELOID_DIFF_TF | 2.11 |
| OXSTRESS_RPE_HNETBH_DN | 2.09 |
| HIVNEFPATHWAY | 2.08 |
| OXSTRESS_BREASTCA_UP | 2.07 |
| HBX_HCC_DN | 2.05 |
| BCNU_GLIOMA_MGMT_24HRS_DN | 2.05 |
| HSA04920_ADIPOCYTOKINE_SIGNALING_PATHWAY | 2.04 |
| GALE_FLT3ANDAPL_DN | 2.03 |
| HDACI_COLON_CLUSTER9 | 2.02 |
| BYSTROM_IL5_UP | 2.02 |
| IL1RPATHWAY | 2.02 |
| HSA05221_ACUTE_MYELOID_LEUKEMIA | 2.01 |
| AD12_32HRS_DN | 1.98 |
| INOS_ALL_DN | 1.98 |
| UVC_TTD_ALL_UP | 1.97 |
| ET743_RESIST_UP | 1.97 |
| DEATHPATHWAY | 1.96 |
| HOFMANN_MANTEL_LYMPHOMA_VS_LYMPH_NODES_DN | 1.96 |
| HSA04210_APOPTOSIS | 1.95 |
| UVC_TTD_4HR_UP | 1.95 |
| UVB_NHEK3_C6 | 1.95 |
| FERRANDO_MLL_T_ALL_UP | 1.93 |
| TGZ_ADIP_UP | 1.93 |
| AD12_ANY_DN | 1.93 |
| HSA00251_GLUTAMATE_METABOLISM | 1.92 |
| CERAMIDEPATHWAY | 1.90 |
| TNFA_NFKB_DEP_UP | 1.90 |
| KLEIN_PEL_UP | 1.89 |
| UV_ESR_OLD_UNREG | 1.89 |
| GH_EXOGENOUS_LATE_UP | 1.89 |
| HSC_LATEPROGENITORS_ADULT | -1.89 |
| HSC_LATEPROGENITORS_SHARED | -1.89 |
| HIPPOCAMPUS_DEVELOPMENT_PRENATAL | -1.89 |
| HDACI_COLON_SUL16HRS_DN | -1.89 |
| HSA04120_UBIQUITIN_MEDIATED_PROTEOLYSIS | -1.89 |
| PEART_HISTONE_UP | -1.90 |
| ZHAN_MMPC_SIM | -1.90 |
| HDACI_COLON_BUT16HRS_DN | -1.90 |
| HDACI_COLON_BUT48HRS_DN | -1.90 |
| MTORPATHWAY | -1.90 |
| ABRAHAM_MM_VS_AL_UP | -1.91 |
| ROSS_FAB_M7 | -1.91 |
| AGED_MOUSE_CORTEX_UP | -1.91 |
| LEE_TCELLS1_UP | -1.91 |
| LEE_TCELLS10_UP | -1.91 |
| LEE_TCELLS8_UP | -1.91 |
| MMS_MOUSE_LYMPH_HIGH_4HRS_UP | -1.92 |
| PARK_HSC_VS_MPP_UP | -1.92 |
| MOREAUX_TACI_HI_IN_PPC_UP | -1.92 |
| SRC_ONCOGENIC_SIGNATURE | -1.92 |
| AGUIRRE_PANCREAS_CHR9 | -1.92 |
| NOUZOVA_CPG_H4_UP | -1.92 |
| HSA05218_MELANOMA | -1.93 |
| 4NQO_ESR_WS_UNREG | -1.93 |
| CMV_HCMV_TIMECOURSE_ALL_DN | -1.93 |
| ABRAHAM_AL_VS_MM_DN | -1.94 |
| HSA04730_LONG_TERM_DEPRESSION | -1.94 |
| DIAB_NEPH_DN | -1.94 |
| UVB_SCC_UP | -1.94 |
| HSC_STHSC_ADULT | -1.94 |
| BYSTRYKH_HSC_CIS_GLOCUS | -1.94 |
| CARIES_PULP_DN | -1.95 |
| SANA_IFNG_ENDOTHELIAL_DN | -1.95 |
| HSA00130_UBIQUINONE_BIOSYNTHESIS | -1.95 |
| MARCINIAK_CHOP_DIFF | -1.95 |
| CELL_CYCLE_REGULATOR | -1.96 |
| HSA04720_LONG_TERM_POTENTIATION | -1.96 |
| FALT_BCLL_IG_MUTATED_VS_WT_DN | -1.96 |
| TPA_SENS_EARLY_DN | -1.96 |
| PROTEASOME | -1.97 |
| ZHAN_MM_CD1_VS_CD2_DN | -1.97 |
| SMITH_HCV_INDUCED_HCC_UP | -1.97 |
| PROTEASOME_DEGRADATION | -1.97 |
| HPV31_UP | -1.98 |
| POMEROY_DESMOPLASIC_VS_CLASSIC_MD_UP | -1.99 |
| PROTEASOMEPATHWAY | -1.99 |
| BRCA1_OVEREXP_PROSTATE_UP | -1.99 |
| HSA03050_PROTEASOME | -2.00 |
| CHESLER_BRAIN_CIS_GENES | -2.00 |
| HSC_STHSC_FETAL | -2.00 |
| HSC_STHSC_SHARED | -2.00 |
| AGED_MOUSE_HIPPOCAMPUS_MULTI_UP | -2.01 |
| MOREAUX_TACI_HI_VS_LOW_DN | -2.01 |
| CHESLER_HIGHEST_FOLD_RANGE_GENES | -2.01 |
| HEARTFAILURE_ATRIA_DN | -2.03 |
| AGEING_LYMPH_DN | -2.05 |
| CALRES_MOUSE_DN | -2.05 |
| UVC_LOW_ALL_DN | -2.05 |
| CROMER_HYPOPHARYNGEAL_MET_VS_NON_UP | -2.05 |
| IDX_TSA_DN_CLUSTER1 | -2.05 |
| LEE_MYC_TGFA_UP | -2.05 |
| AGUIRRE_PANCREAS_CHR12 | -2.06 |
| GAMMA-UV_FIBRO_UP | -2.07 |
| ROME_INSULIN_2F_UP | -2.07 |
| SASAKI_ATL_UP | -2.08 |
| SASAKI_TCELL_LYMPHOMA_VS_CD4_UP | -2.08 |
| AGED_MOUSE_CORTEX_DN | -2.08 |
| G_PROTEIN_SIGNALING | -2.11 |
| HDACI_COLON_SUL12HRS_DN | -2.11 |
| UVC_LOW_C3_DN | -2.11 |
| PRMT5_KD_UP | -2.13 |
| LEE_MYC_UP | -2.14 |
| AGED_MOUSE_HYPOTH_UP | -2.14 |
| TSADAC_HYPERMETH_OVCA_UP | -2.21 |
| H2O2_CSBDIFF_C1 | -2.22 |
| IFNALPHA_RESIST_DN | -2.25 |
| HSA00563_GLYCOSYLPHOSPHATIDYLINOSITOL_ANCHOR_BIOSYNTHESIS | -2.26 |
| BRCA1_OVEREXP_UP | -2.28 |
| FLECHNER_KIDNEY_TRANSPLANT_REJECTION_PBL_UP | -2.30 |
| HOGERKORP_CD44_DN | -2.31 |

**Table S7.** Molecular networks representing the recurrence-free survival gene expression signature (91 genes) in HCC patients with late (>1 year) recurrence.

| Rank | Score* | Focus molecules | Molecular functions |
| --- | --- | --- | --- |
| 1 | 25 | 14 | Cancer, Embryonic Development, Reproductive System Disease |
| 2 | 18 | 11 | Cellular Assembly and Organization, Cellular Function and Maintenance, Molecular Transport |
| 3 | 18 | 11 | Cell Cycle, Cancer, Antimicrobial Response |
| 4 | 2 | 1 | Carbohydrate Metabolism, Nucleic Acid Metabolism, Small Molecule Biochemistry |
| 5 | 2 | 1 | Carbohydrate Metabolism, Digestive System Development and Function, Hair and Skin Development and Function |
| 6 | 2 | 1 | Cell Morphology, Embryonic Development, Cellular Assembly and Organization |
| 7 | 2 | 1 | Connective Tissue Disorders, Genetic Disorder, Immunological Disease |
| 8 | 2 | 1 | Cancer, Immunological Disease, Cell Death |
| 9 | 2 | 1 | Cell Morphology, Cellular Compromise, Molecular Transport |
| 10 | 2 | 1 | Cellular Function and Maintenance, Cellular Development, Cellular Growth and Proliferation |
| 11 | 2 | 1 | Cell Signaling, Developmental Disorder, Genetic Disorder |
| 12 | 2 | 1 | Cell Signaling, Cell Morphology, Embryonic Development |
| 13 | 2 | 1 | Post-Translational Modification, Protein Folding, Dermatological Diseases and Conditions |
| 14 | 2 | 1 | Cell Morphology, Post-Translational Modification, Cellular Assembly and Organization |

The recurrence-free survival gene expression signature (91 genes) in HCC patients with late (>1 year) recurrence was analyzed with Ingenuity software.

*Ingenuity-derived significance score for the molecular network.
