# Supplementary material for: Gene expression in nontumoral liver tissue and recurrence-free survival in hepatitis C virus-positive hepatocellular carcinoma
Source: Mol Cancer. 2010 Apr 9;9:74. doi: 10.1186/1476-4598-9-74 (PMC2856554; doi:10.1186/1476-4598-9-74)
Supplement: Additional file 2 — Figure S1. TGFβ1- and MYC-centered interactomes are most significantly up-regulated molecular networks in HCC tumors (see Additional file 1, Table S2-3 for a full list of the significant pathways). Ellipse, square, triangle, trapezoid, lozenge and circle represent transcription regulator, cytokine, kinase, transporter, enzyme and other molecules, respectively. Red and white represent molecules up-regulated in tumor samples compared to control and molecules incorporated into the network, respectively. Arrows connecting molecules indicate one molecule acts on another and lines indicate one binds to another. Dashed arrows or lines indicate indirect interactions of two molecules. Figure S2. Analysis of the dissimilarity (Spearman's correlation) between gene expression profiles of the nontumoral (top) or tumoral (bottom) samples used in this study. Expression of 25,073 genes was used as a variable to create the heat map of each dissimilarity matrix. The color bar represents the degree of dissimilarity with red exhibiting high degree of dissimilarity and blue - high similarity. Figure S3. Recurrence-free survival analysis in HCC patients using gene set analysis in tumor samples. (A) Logrank P-value and error rate of prediction for up to 150 top-ranked gene sets. (B) Recurrence-free survival curves (Kaplan-Meier method) based of the prediction made using 67 top-ranked gene sets (Additional file 1, Table S4). Red and blue represent the subjects predicted to have poor or good recurrence-free survival, respectively. (C) Heatmap of the 67 gene set-based survival signature from tumor samples. Red and blue indicate high or low gene set score, respectively. Subjects (x-axis) are sorted by means of cosine distance to 'poor' template, and gene sets (y-axis) are sorted by Cox score (Additional file 1, Table S4). Figure S4. Recurrence-free survival analysis in HCC patients with late (>1 year) recurrence using gene expression (91 genes) in nontumoral samples. (A) 91 genes selected bas [file 1476-4598-9-74-S2.DOC]

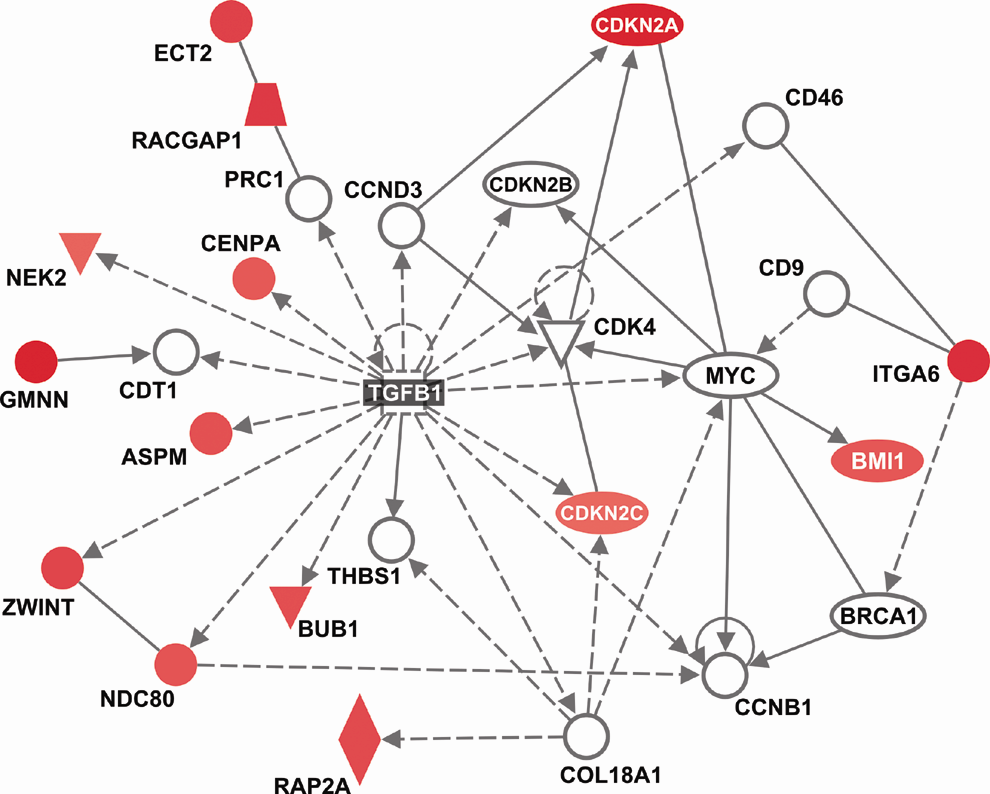


**Figure S1.** TGFβ1- and MYC-centered interactomes are most significantly up-regulated molecular networks in HCC tumors (see Supplemental Table S2-3 for a full list of the significant pathways). Ellipse, square, triangle, trapezoid, lozenge and circle represent transcription regulator, cytokine, kinase, transporter, enzyme and other molecules, respectively. Red and white represent molecules up-regulated in tumor samples compared to control and molecules incorporated into the network, respectively. Arrows connecting molecules indicate one molecule acts on another and lines indicate one binds to another. Dashed arrows or lines indicate indirect interactions of two molecules.


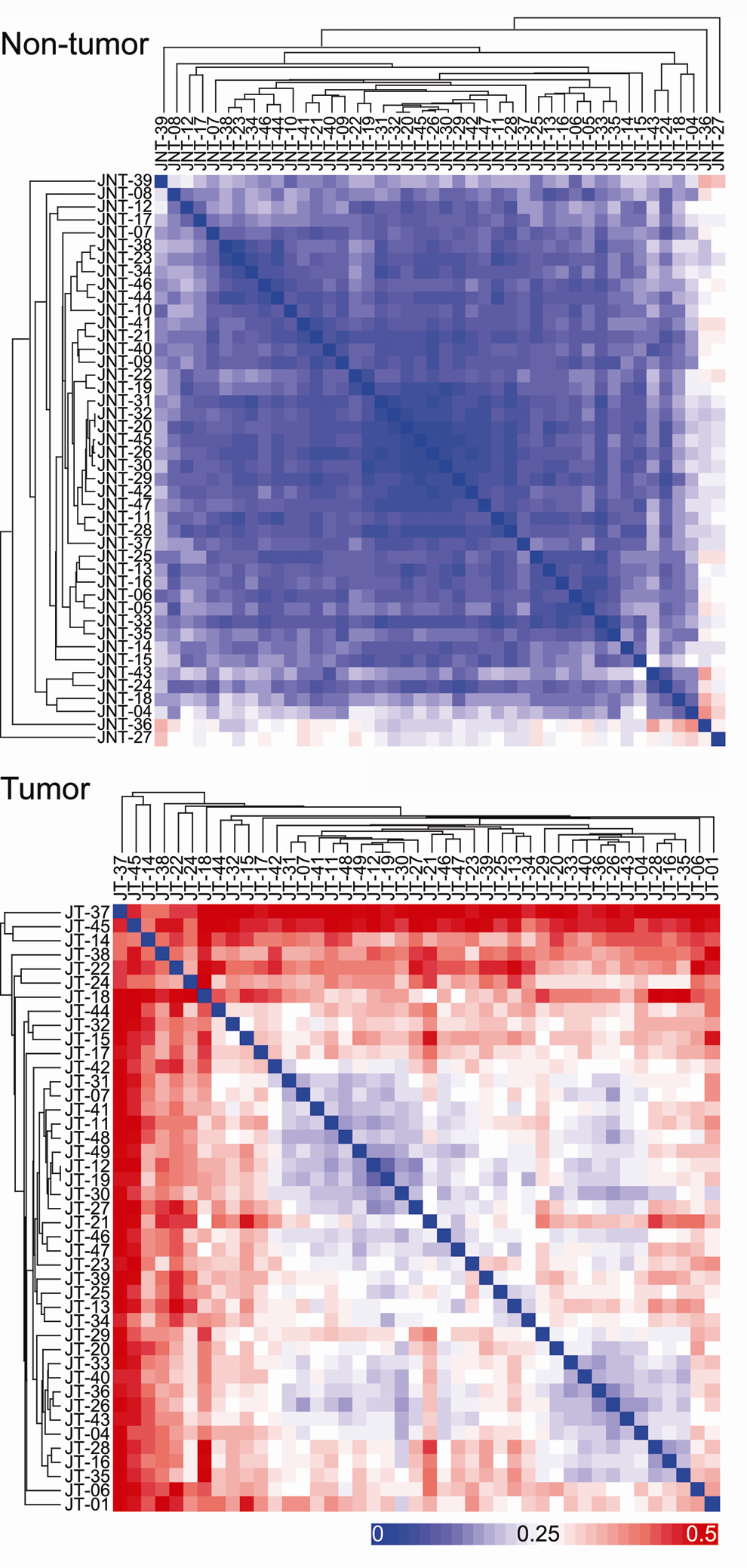


**Figure S2.** Analysis of the dissimilarity (Spearman’s correlation) between gene expression profiles of the nontumoral (top) or tumoral (bottom) samples used in this study. Expression of 25,073 genes was used as a variable to create the heat map of each dissimilarity matrix. The color bar represents the degree of dissimilarity with red exhibiting high degree of dissimilarity and blue – high similarity.


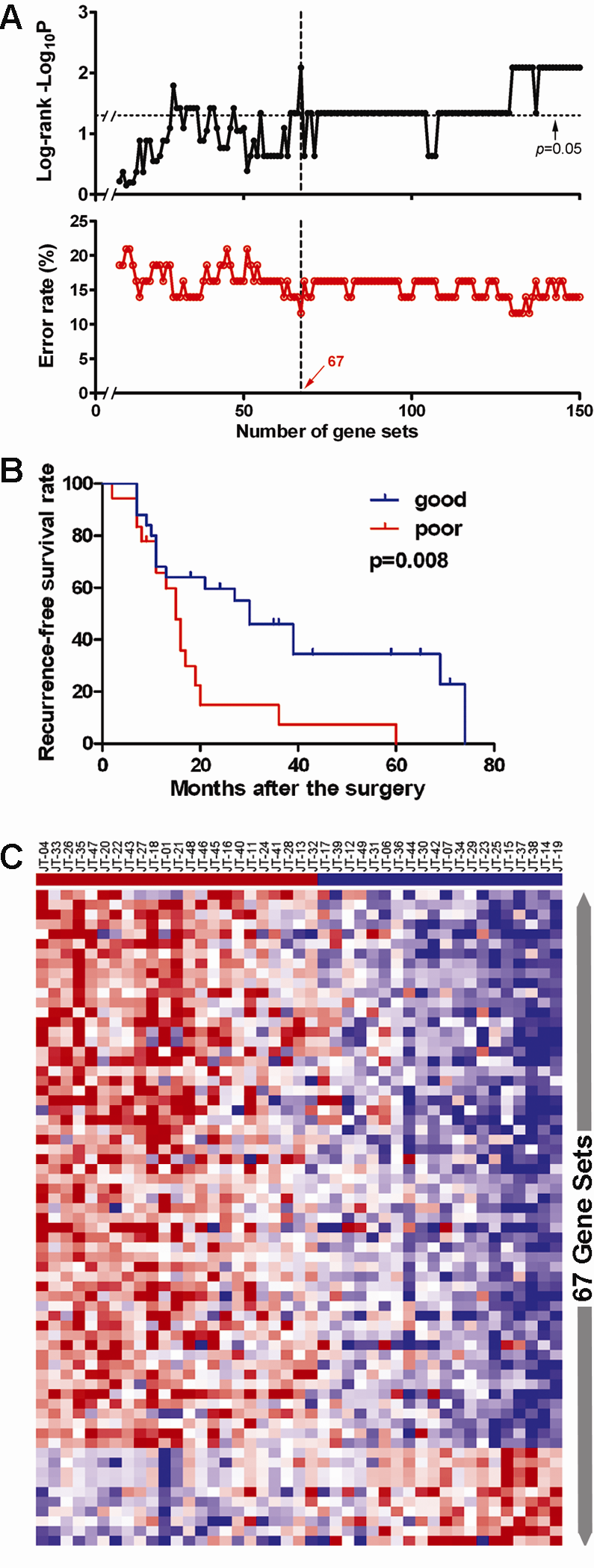


**Figure S3.** Recurrence-free survival analysis in HCC patients using gene set analysis in tumor samples. (A) Logrank P-value and error rate of prediction for up to 150 top-ranked gene sets. (B) Recurrence-free survival curves (Kaplan-Meier method) based of the prediction made using 67 top-ranked gene sets (Supplemental Table S4). Red and blue represent the subjects predicted to have poor or good recurrence-free survival, respectively. (C) Heatmap of the 67 gene set-based survival signature from tumor samples. Red and blue indicate high or low gene set score, respectively. Subjects (x-axis) are sorted by means of cosine distance to ‘poor’ template, and gene sets (y-axis) are sorted by Cox score (Supplemental Table S4).


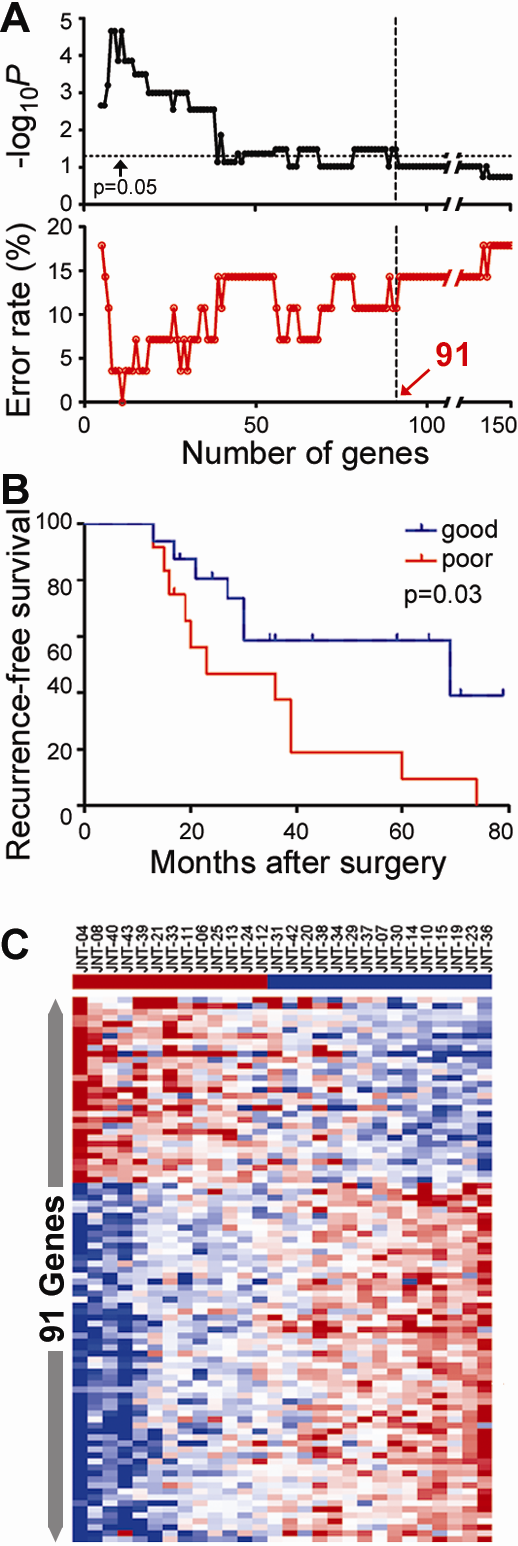


**Figure S4.** Recurrence-free survival analysis in HCC patients with late (>1 year) recurrence using gene expression (91 genes) in nontumoral samples. (A) 91 genes selected based on log-rank P-value and error rate of prediction were used to construct recurrence-free survival curves (B). (C) Heat map of the gene-based survival signature with 91 genes.
